# Acute mechanical stress in primary porcine RPE cells induces angiogenic factor expression and in vitro angiogenesis

**DOI:** 10.1186/s13036-020-00235-4

**Published:** 2020-04-25

**Authors:** Farhad Farjood, Amir Ahmadpour, Sassan Ostvar, Elizabeth Vargis

**Affiliations:** 1 Department of Biological Engineering, Utah State University, 4105 Old Main Hill, Logan, UT 84322 USA; 2 Present address: Neural Stem Cell Institute, Rensselaer, NY 12144 USA; 3 Present address: Department of Animal Sciences, Yasouj University, Yasouj, 75918-74934 Iran; 4 Division of General Medicine, Columbia University Medical Center, New York, NY 10032 USA

**Keywords:** Mechanical stress, Angiogenesis, RPE, AMD, EMT, CNV, VEGF, IL-6, IL-8, ANG2

## Abstract

**Background:**

Choroidal neovascularization (CNV) is a major cause of blindness in patients with age-related macular degeneration. CNV is characterized by new blood vessel growth and subretinal fluid accumulation, which results in mechanical pressure on retinal pigment epithelial (RPE) cells. The overexpression of RPE-derived angiogenic factors plays an important role in inducing CNV. In this work, we investigated the effect of mechanical stress on the expression of angiogenic factors in porcine RPE cells and determined the impact of conditioned medium on in-vitro angiogenesis.

**Results:**

The goal of this study was to determine whether low levels of acute mechanical stress during early CNV can induce the expression of angiogenic factors in RPE cells and accelerate angiogenesis. Using a novel device, acute mechanical stress was applied to primary porcine RPE cells and the resulting changes in the expression of major angiogenic factors, VEGF, ANG2, HIF-1α, IL6, IL8 and TNF*-*α, were examined using immunocytochemistry, qRT-PCR, and ELISA. An in vitro tube formation assay was used to determine the effect of secreted angiogenic proteins due to mechanical stress on endothelial tube formation by human umbilical vein endothelial cells (HUVECs). Our results showed an increase in the expression of VEGF, ANG2, IL-6 and IL-8 in response to mechanical stress, resulting in increased in vitro angiogenesis. Abnormal epithelial-mesenchymal transition (EMT) in RPE cells is also associated with CNV and further retinal degeneration. Our qRT-PCR results verified an increase in the expression of EMT genes, CDH2, VIM and FN1, in RPE cells.

**Conclusions:**

In conclusion, we showed that acute mechanical stress induces the expression of major angiogenic and EMT factors and promotes in vitro angiogenesis, suggesting that mechanical stress plays a role in promoting aberrant angiogenesis in AMD.

## Background

The choroid is a vascular layer underneath the retinal pigment epithelium (RPE) that supplies blood to the RPE and the retina. The abnormal overgrowth of choroidal blood vessels creates a condition called choroidal neovascularization (CNV). In age-related macular degeneration (AMD), CMV damages the overlying RPE and photoreceptors, resulting in irreversible blindness. The etiology of CNV remains to be fully outlined, but RPE cells do produce higher levels of angiogenic proteins in response to mechanical stress, promoting angiogenesis and contributing to CNV development [1, 2]. Choroidal blood vessel invasion and sub-RPE fluid accumulation are potential sources of mechanical stress during AMD. As new blood vessels form, creating spatial crowding and hemorrhages, RPE cells elongate from < 10% to ~ 60%, uniaxially [3–5]. However, little is known about the resulting angiogenic factor expression in RPE cells.

Many pro-angiogenic proteins are involved in CNV, including the RPE-derived vascular endothelial growth factor (VEGF), angiopoietin 2 (ANG2), and fibroblast growth factor 2 (FGF2) [6–8]. Interleukin-6 (IL-6), interleukin-8 (IL-8) and tumor necrosis factor-α (TNF-α), are also involved in choroidal angiogenesis [9–11]. Another marker of CNV pathogenesis is epithelial-mesenchymal transition (EMT), which promotes RPE de-differentiation [12, 13], and is triggered by angiogenic cytokines, such as TNF-α, VEGF, Il-6 and IL-8 [14–17]. Mechanical stress may promote EMT in RPE cells by inducing the expression of these factors.

To test whether increased mechanical stress on RPE cells during early stages of CNV accelerates CNV development, we modified an in vitro technique [2] to model low levels of strain in the RPE (10% uniaxial strain) to mimic those experienced during early CNV. Then, we studied how mechanical stress effects mRNA and protein expression of major angiogenic factors: VEGF, ANG2, hypoxia-inducible factor-1α (HIF-1α), IL-6, IL-8, and TNF-α; and EMT markers: vimentin (VIM), cadherin 2 (CDH2), and fibronectin-1 (FN1). In addition, we used finite element analysis and immunocytochemistry to find correlations between strain levels and increased expression of VEGF, IL-6 and IL-8. We also assessed the angiogenic potential of the stress-induced RPE secretome using an in vitro angiogenesis assay with human umbilical vein endothelial cells (HUVECs). HUVECs are capable of forming endothelial tube-like networks, similar to microvasculature, when cultured on 3D basement membranes and, therefore, have been widely used to study angiogenesis in different experimental settings [18, 19]. We employed HUVECs to evaluate the angiogenesis potential of the conditioned media from RPE cultures on in vitro angiogenesis. Our results showed that mechanical stress similar to that experienced during early CNV induces the expression of angiogenic and EMT factors in RPE cells, further promoting in vitro angiogenesis.

## Results

### Characterization of isolated porcine RPE cells

Isolated porcine RPE cells showed characteristics of differentiated RPE after 4 weeks of culture on Transwell membranes. Confocal images showed adherens junction protein, β-catenin (Fig. 2a), F-actin (Fig. 2b), and tight junction protein, ZO-1 (Fig. 2c) along the cell-cell junctions, as well as the cytoplasmic expression of the RPE-specific protein, RPE65 (Fig. 2d). The typical RPE cobblestone morphology was also observed in brightfield images of 4-week-old cultures (Fig. 2e). The TEER values increased from 65.38 to 767.05 Ω.cm^2^ (*p* = 1.46 E-6) after 4 weeks (Fig. 2f).

### Increased strain leads to increased VEGF, IL-6, and IL-8 expression

Finite element analysis showed that the mechanically stressed area’s center experiences the highest strain level (Fig. 3a). These results were compared to the ICC results to determine whether strain levels and increased angiogenic protein expression are correlated.

Confocal images of mechanically-stressed RPE cells showed that in non-stressed cells, F-actin localized to cell-cell junctions (Fig. 2b), while in mechanically stressed cells, the actin filaments distributed diffusely in the cytoplasm (Fig. 3c, h, m). ICC results also showed that the mechanical stress-induced disruption of the F-actin cytoskeleton was associated with increases in VEGF (Fig. 3b-e), IL-6 (Fig. 3g-j), and IL-8 (Fig. 3l-o) expression. The RPE monolayer also began to deform after stretching the Transwell membranes, based on z-stack images and (Fig. 3f, k, p), as predicted by FEA (Fig. 3a).

### Mechanical stress in RPE cells induces the expression of angiogenic and EMT factors and promotes in vitro angiogenesis

qRT-PCR results showed substantial changes in the expression of major angiogenic and EMT mRNA (Fig. 3q, r). Three hours after applying mechanical stress, the expression of angiogenic factors, *VEGF121*, *HIF-1α*, *ANG2* and *IL-8*, increased significantly (*p* = 0.018, 0.007, 0.021, 0.018, respectively). The expression of pigment epithelium-derived factor (PEDF), an anti-angiogenic gene, was also increased (*p* = 0.044). We observed a significant increase in the expression of the EMT promoter, *TNF-α* (*p* = 0.005). After 6 hours, the expression of two major *VEGF* isoforms, *VEGF121* and *VEGF165,* and a stimulator of *VEGF* expression, *HIF-1α,* increased (*p* = 0.011, 0.016, 0.011, respectively). Moreover, the expression of *ANG2*, *IL-6* and *IL-8*, *PEDF* and *TNF-α* also increased significantly (*p* = 0.017, 0.011, 0.013, 0.013, 0.016, respectively; Fig. 3q). While *FGF2* gene expression in mechanically stressed RPE cells decreased significantly after 6 hours (*p* = 0.017), there was no significant change in apical and basal FGF2 protein expression (*p* = 0.52, 0.49, respectively; Fig. 3q, s, t).

We also observed a significant increase in the following EMT markers, *CDH2* (p (3 h) = 0.044, p (6 h) = 0.024), *VIM* (p (3 h) = 0.094, p (6 h) = 0.017), and *FN1* (p (3 h) = 0.044, p (6 h) = 0.017), and a decrease in *CDH1* (p (3 h) = 0.044, p (6 h) = 0.051) and an RPE-specific gene, *RPE65* (p (3 h) = 0.03, p (6 h) = 0.00; Fig. 3r). ELISA results showed a significant increase in the expression of IL-6, apically (*p* = 0.014 respectively), and VEGF, ANG2, IL-6 and IL-8, basolaterally (*p* = 0.042, 0.008, 0.000, 0.019 respectively; Fig. 3s, t).

qRT-PCR results showed a concurrent increase in pro-angiogenic gene expression, as mentioned above, as well as the anti-angiogenic factor, *PEDF* (Fig. 3q). To determine whether the increase of pro-angiogenic factors overrides the anti-angiogenic activity of PEDF, an in vitro angiogenesis assay was performed. Conditioned media from RPE cultures activated endothelial tube formation, while limited angiogenic activity was observed in HUVECs cultured in fresh RPE media (Fig. 4). Exposure to apical and basal conditioned media from mechanically stressed RPE cells resulted in a significant ~ 2- and 1.4-fold increase in endothelial tube length (*p* = 0.001, 0.017, respectively) and ~ 3.6- and 2-fold increase in node number respectively (*p* = 0.003, 0.018, respectively; Fig. 4f, g).

## Discussion

**Fig. 1:**
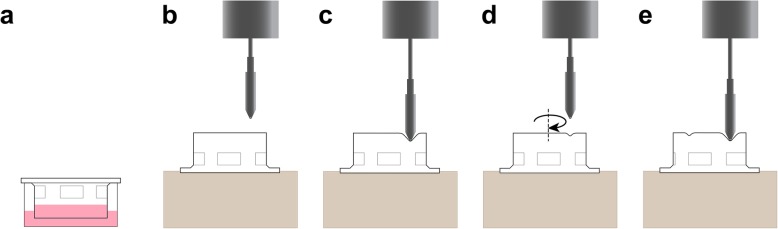
Schematic of the mechanical stress device. RPE cells were grown on porous membranes of Transwell inserts (**a**). After 4 weeks, Transwells were placed upside down on a custom-made stage under a pen tip and controlled with a rotor (**b**). The pen tip was pushed against the Transwell membrane to permanently stretch focal regions of the membrane (**c**). The Transwell insert was rotated between impacts to produce 60 non-overlapping bumps in 2 min (approximately 2 s per impact) (**d**, **e**). The black boxes correspond to the perforated areas of the Transwell inserts and the inserts’ rotation is indicated following step d”

While the mechanisms leading to increased expression of RPE-derived angiogenic factors and the resulting angiogenesis during CNV in AMD are not entirely clear, physical changes in the RPE contribute to the elevated expression of angiogenic factors [1, 2, 20]. To better understand the role that mechanical stress plays in AMD pathogenesis, we investigated the effect of mechanical stress on the expression of angiogenic and EMT factors and in vitro angiogenesis using a novel in vitro device (Fig. 1).

**Fig. 2:**
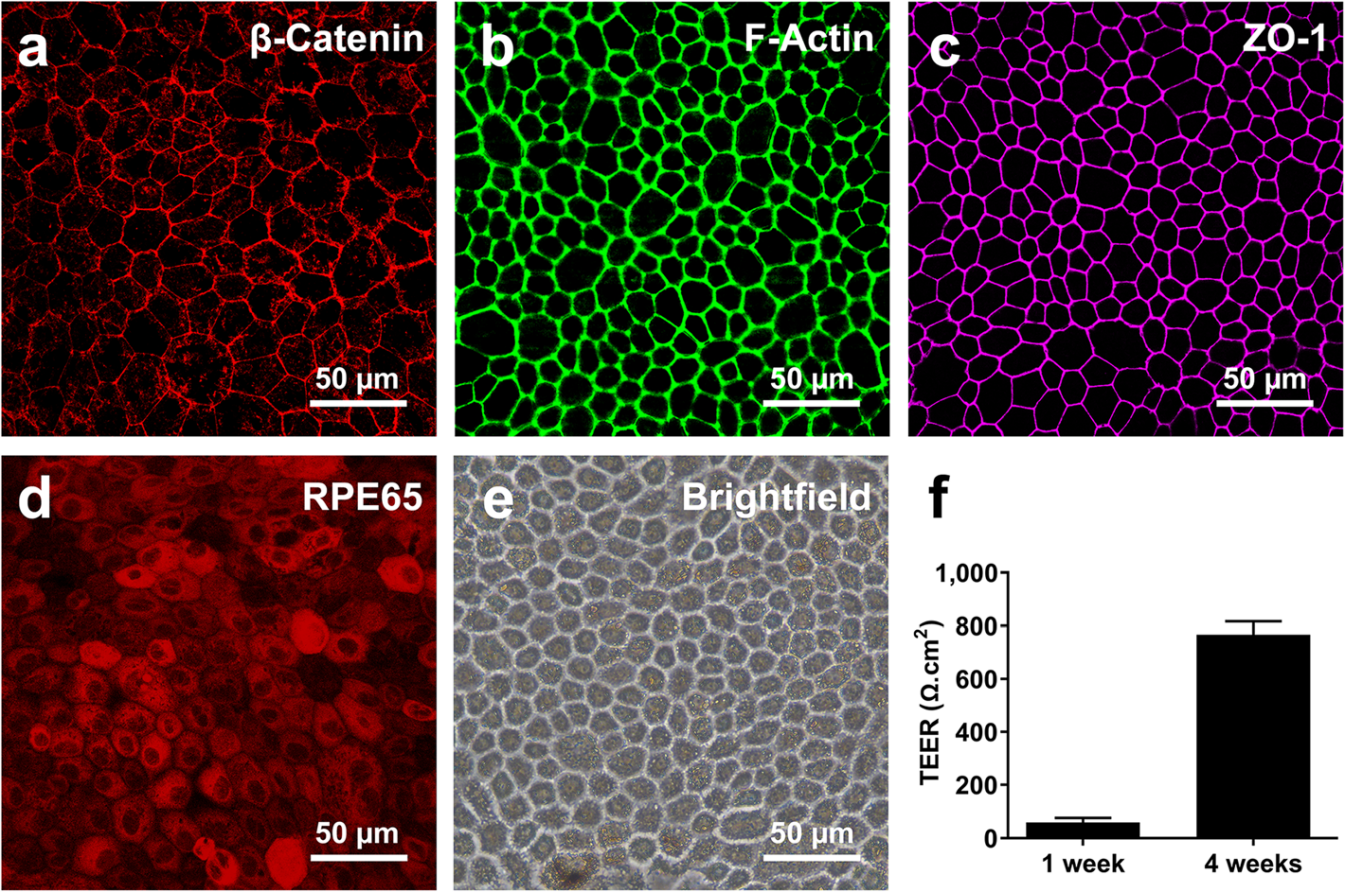
Characterization of porcine RPE monolayers. ICC results confirm the proper localization of β-catenin (**a**), F-actin (**b**), and ZO-1 (**c**) and the expression of RPE65 (**d**) in isolated RPE cells. Brightfield imaging showed the characteristic cobblestone morphology of RPE cells (**e**). TEER values reached ~ 767 Ω.cm^2^ after 4 weeks, indicating maturation of the RPE and establishment of an in vivo-like blood-retinal barrier (**f**). Error bars represent one standard deviation

Mechanical stress in many cell types, including periodontal ligament fibroblasts (HPLF), mesenchymal stem cells and HUVECs, has been found to affect angiogenesis [21–23]. ARPE-19 cells, a human RPE cell line, express increased levels of angiogenic cytokines, such as VEGF and IL6, involved in various retinal diseases in response to cyclic mechanical stress [1, 24]. However, ARPE-19 cells lack important RPE characteristics, such as RPE-specific marker expression and high transepithelial resistance [25]. A more realistic RPE model is needed to characterize the effect of mechanical stress-derived changes to the RPE secretome and angiogenesis. To this end, we produced a realistic in vitro model of the RPE by growing freshly isolated porcine RPE cells on Transwell membranes. Porcine RPE has been used previously as a realistic in vitro model to study many RPE functions, including VEGF expression and in vitro angiogenesis, demonstrating its suitability for the purpose of this study [26–28]. Our in vitro porcine RPE cultures demonstrated several characteristics similar to native RPE, such as junctional localization of β-catenin, F-actin, and ZO-1, expression of the RPE-specific RPE65, and high TEER in 4-week-old RPE cultures (Fig. 2), indicating that the substrate supports proper maturation of RPE cells. Next, using a novel device, mechanical stress was added to the RPE monolayer to determine changes in gene and protein expression of major angiogenic and EMT factors. Lastly, an in vitro angiogenesis assay was performed to determine if pro-angiogenic factor production outweighed anti-angiogenic factor production and induced angiogenesis.

**Fig. 3:**
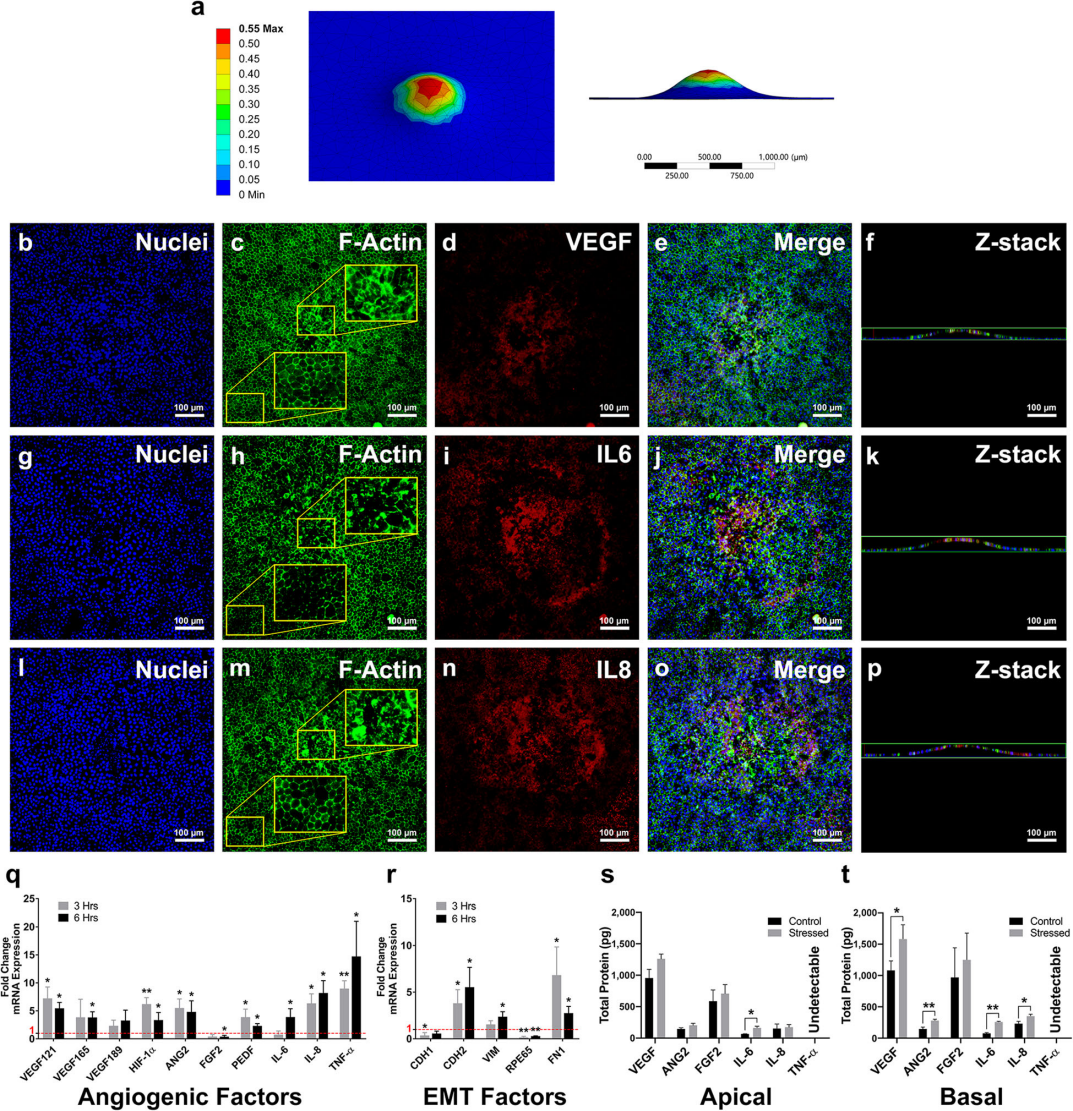
Mechanical elongation of the RPE induces the expression of angiogenic, inflammatory and EMT genes and proteins. **(a)** Mechanically stressing the Transwell membrane with our device results in plastic deformation of the membrane with the highest strain occurring in the center of the stressed area. (**b-p)** Confocal images of three mechanically stress samples (Sample 1: **b-f**, Sample 2: **g-k**, Sample 3: **l-p**) showed that the expression of VEGF (**d**), IL-6 (**i**) and IL-8 (**n**) increased and F-actin structures were disrupted (**c**, **h**, **m**) due to increased mechanical stress. Z-stack images confirm the deformation of the RPE monolayer after adding mechanical stress (**f**, **k**, **p**). qRT-PCR results show increased expression of *VEGF* isoforms, *VEGF121* and *VEGF165*, *HIF-1α, ANG2, IL-6, IL-8, TNF-α* and the antiangiogenic factor, *PEDF* (**q**). An increase in the expression of EMT genes, *VIM* and *CDH2* and fibrosis gene, *FN1*, and a decrease in the expression of RPE-specific *RPE65* was also observed (**r**). ELISA results showed increased apical expression of IL-6 and basal expression of VEGF, ANG2, IL-6 and IL-8 (**s**, **t**). * *p* < 0.05 ** *p* < 0.01. Error bars represent one standard deviation

Our results showed elevated mRNA expression of angiogenic factors, VEGF, HIF-1α, IL-6, IL-8, ANG2, and anti-angiogenic PEDF (Fig. 3q), and increased protein expression of VEGF, IL-6, IL-8, and ANG2 in mechanically stressed RPE cells (Fig. 3b-p, s, t). ICC results also revealed a remarkable disruption of the actin cytoskeleton in RPE sites with higher VEGF, IL-6 and IL-8 expression. The stressed areas of the RPE showed diffusely distributed actin fibers, while in the non-stressed areas, actin was localized in cell-cell junctions (insets in Fig. 3c, h, m). According to the FEA (Fig. 3a), in these areas of impact, strain distribution pattern correlated with the disruption of actin structure and increased VEGF, IL-6, and IL-8 expression, supporting the hypothesis that the increase in mechanical stress is responsible for elevated angiogenic factor expression.

The increase in IL-8 and VEGF expression (Fig. 3q, s, t) was consistent with previous reports of IL-8 and VEGF induction in human RPE cells after RPE injury [29, 30]. It has previously been shown that actin polymerization activation induces IL-8 expression and different *VEGF* isoforms [31, 32] . It is therefore possible that disrupting the actin cytoskeleton activates actin polymerization, leading to the increased expression of IL-8 and different *VEGF* isoforms. Unlike *VEGF121* and *VEGF165*, *VEGF189* was not sensitive to the mechanical stress levels used in our experiments (Fig. 3q). The two soluble *VEGF* isoforms, *VEGF121* and *VEGF165*, are regulated by low frequency stress while insoluble *VEGF189* is more susceptible to high frequency mechanical stress [32]. The single pulse of mechanical stress used in our experiment may be lower than required for *VEGF189* mRNA overexpression. Both soluble VEGF isoforms, VEGF121 and VEGF165, have been implicated in in vitro and in vivo neovascularization [33–36]. Hence, their overexpression induced by mechanical stress may contribute to CNV development.

We also observed an increase in the expression of *HIF-1α* in response to mechanical stress (Fig. 3q). HIF-1α is an inducer of VEGF, IL-6, and IL-8 under hypoxic conditions [37, 38], and its mRNA expression was activated by mechanical stress, suggesting that the mechanisms of mechanical stress-induced VEGF, IL-6, and IL-8 overexpression may be similar to hypoxic conditions. Further investigation is needed to accurately assess the involvement of HIF-1*α* in inducing angiogenic factors in RPE cells due to mechanical stress.

Our gene expression results also showed an increase in the expression of *TNF-α* in response to mechanical stress (Fig. 3q). Mechanical stress can induce oxidative stress in RPE cells, which in turn activates TNF-α transcription [39, 40]. Our qRT-PCR results confirm these previous findings by implicating mechanical stress in inducing TNF*-α* gene expression. However, ELISA results showed undetectably low levels of TNF-α in RPE supernatants before and after applying mechanical stress, suggesting that the low level of mechanical stress used in this study may not be enough to activate TNF-α protein expression in RPE cells. Further experiments with different mechanical stress levels will elucidate the mechanism of mechanical stress-induced TNF-α expression in RPE cells and explain the lack of TNF-α protein expression despite increased transcription.

The qRT-PCR results showed that mechanical stress promoted an EMT-like phenotype in RPE cells, as demonstrated by an increase in the expression of mesenchymal markers, *CDH2*, *VIM*, and *FN1*, and a decrease in the expression of the RPE-specific *RPE65* (Fig. 3r). Previous studies have shown that VEGF, IL-6 and IL-8 can trigger EMT [15–17]. Our experiments demonstrated that mechanical stress induced all three of these cytokines, confirming the hypothesis that mechanical stress may induce EMT in the RPE through the induction of EMT promoters during stages of CNV development.

**Fig. 4:**
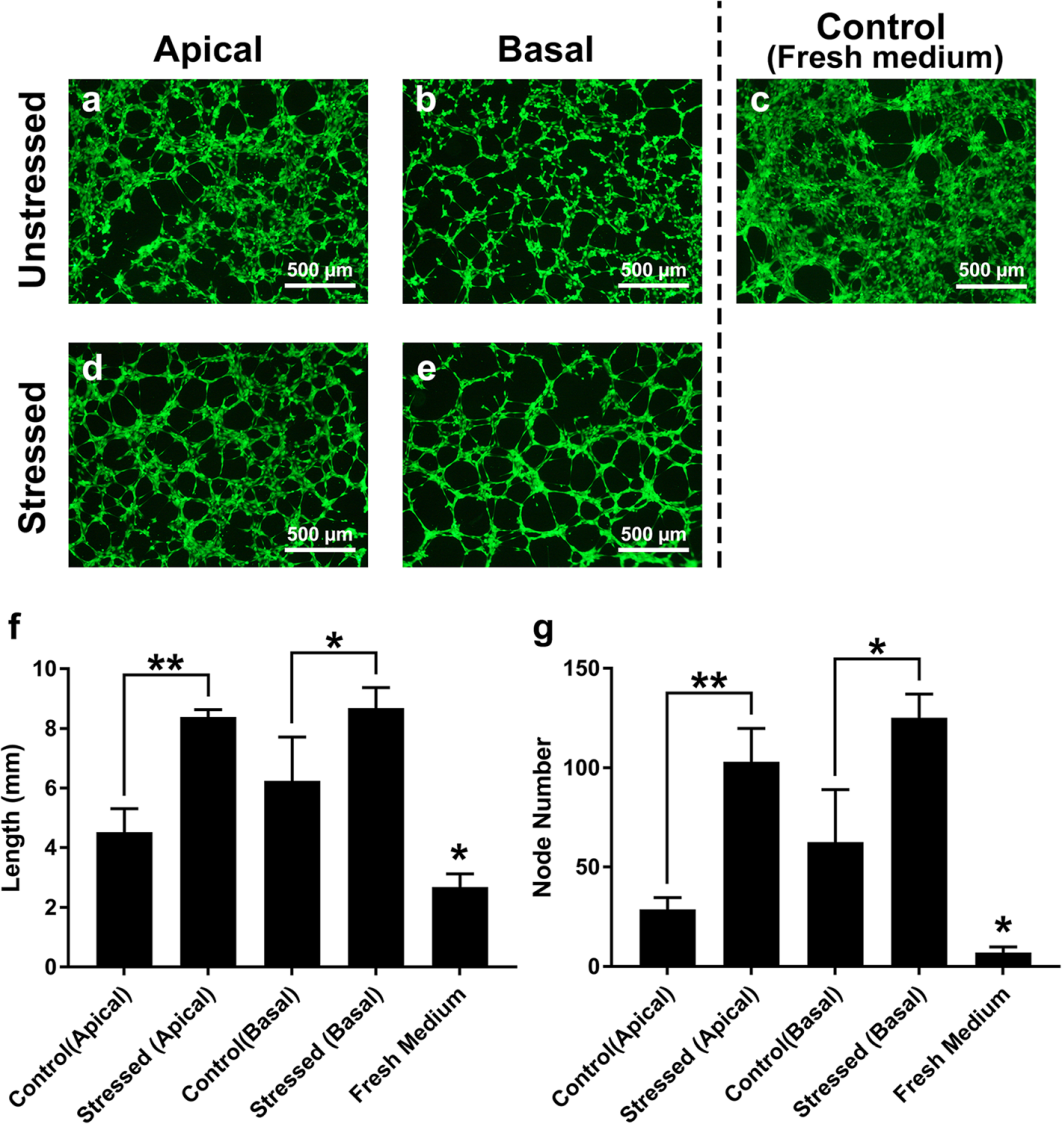
In vitro angiogenesis results. Mechanical stress increased the endothelial tube formation response of HUVECs to used media from RPE cultures. The length of the endothelial tubes and the number of nodes increased when HUVECs were exposed to conditioned apical (**a**, **d**) and basal (**b**, **e**) media from mechanically stressed RPE cultures for 6 h (**f**, **g**). HUVECs incubated with fresh medium resulted in smaller endothelial tubes and fewer nodes compared to those grown with media from both unstressed and mechanically stressed RPE cultures (**c**, **f**, **g**). * *p* < 0.05; ** *p* < 0.01; Control groups were compared to all treatment groups. Error bars represent one standard deviation

The expression of *PEDF*, a major anti-angiogenic gene, also increased due to mechanical stress (Fig. 3q). Previous work has shown that the balance between VEGF and PEDF must change for choroidal angiogenesis initiation [41], The in vitro angiogenesis assay determined if pro-angiogenic protein levels outweigh anti-angiogenic factors and promote angiogenesis. The increased angiogenic response of the HUVECs to the mechanically stressed RPE secretome (Fig. 4) suggests that the increase in the PEDF expression was either not sufficient to neutralize the angiogenic activity of the overexpressed angiogenic factors or did not lead to significant protein secretion. However, the higher increase in the angiogenic potential of the apical supernatant compared to that of the basal supernatant (Fig. 4) could be due to the elevated PEDF expression, as the expression of PEDF is mainly basolateral. Further protein expression analyses are needed to better understand the dynamics of the VEGF/PEDF angiogenic switch under mechanical stress. The choroid is adjacent to the basal side of the RPE and our experiments showed that media from both sides of mechanically-stressed RPE cultures increased in vitro angiogenesis (Fig. 4), suggesting that mechanical stress promotes angiogenesis by inducing the overexpression of angiogenic factors. These results deepen our understanding of the role that mechanical stress may play in the initiation and development of CNV and open potential avenues to more effective therapeutic interventions for neovascularization in AMD.

## Conclusions

In this work, we present a novel method of introducing and understanding mechanical changes in the RPE during early stages of CNV development and reported, for the first time, that acute mechanical stress induces the expression of angiogenic and EMT factors. In vitro angiogenesis results confirmed the main hypothesis that mechanical stress in RPE cells can induce angiogenesis. This result suggests that mechanical stretching of the RPE accelerates angiogenesis during CNV.

## Methods

To identify the effect of mechanical stress on the expression of angiogenic cytokines and in vitro angiogenesis, we first cultured porcine RPE cells on Transwell membranes for 4 weeks. Using a novel mechanical stress device, the porous membrane of the Transwell inserts was stretched to apply mechanical stress to the overlying RPE cells. Immunocytochemistry, qRT-PCR and enzyme-linked immunosorbent assay were used to measure gene and protein expression of angiogenic cytokines and EMT markers. Finally, an in vitro angiogenesis assay was used to measure the angiogenic potential of the conditioned media from control and mechanically stressed RPE cultures.

### RPE isolation and culture

RPE cells were isolated from locally-sourced pig eyeballs using a previously described method [42]. Isolated cells (passage 0) were cultured on Dulbecco’s modification of Eagle medium (DMEM) 1x (Corning, Manassas, VA) supplemented with 10% premium grade fetal bovine serum (FBS; VWR, Radnor, PA) on 2.4 mm Transwell inserts (pore size: 0.4 μm, Corning) and incubated at 37 °C, 5% CO_2_ in a humidified incubator until the inserts were confluent (approximately 3–5 days). After reaching confluency, FBS concentration was dropped to 1% and cells were grown on Transwell inserts for 4 weeks to promote RPE differentiation before the experiments.

### Transepithelial electrical resistance (TEER)

TEER was performed using an EVOM2 voltohmmeter (World Precision Instrument, Sarasota, FL) connected to an ENDOHM-24SNAP measurement chamber (World Precision Instrument). TEER of RPE monolayers was measured after 1 week and 4 weeks of culture on 3 Transwell membranes.

### Mechanical stress

We have previously shown that the plastic properties of the porous polyester Transwell membranes can be permanently stretched to convey mechanical stress to adherent cells [2]. In this work, we fabricated a device to convey controlled and localized stress to RPE monolayers instead. The tip of a ballpoint pen was attached to an in-house reciprocating motion mechanism. A Transwell membrane was placed upside down on a custom stage made from a laser-cut acrylic sheet directly under the pen tip and rotated as the reciprocating motion was engaged to simulate 60 non-overlapping impacts (mechanical stresses) on the membrane over 2 min, creating a single pulse of mechanical stress (Fig. 1). The height of the stage was adjusted so that the tip’s pressure created ~ 10% strain, mimicking low levels of strain on the RPE during early stages of CNV.

### Finite element analysis

Finite element analysis (FEA) was performed using Ansys V19.1 to evaluate strain distribution on Transwell membranes due to mechanical stress. Plastic strain in the membranes was simulated by pushing a 3D model of the pen tip (500 μm diameter) on a 10 μm-thick polyester membrane (same as Transwell membranes) to create 10% strain.

### Immunocytochemistry

Immunocytochemistry (ICC) was performed on RPE cells grown on mechanically stressed Transwell membranes using anti-VEGF, anti-IL-6, and anti-IL-8 primary antibodies (Santa Cruz Biotechnology, Santa Cruz, CA). RPE65, zonula occludens-1 (ZO-1), and β-catenin antibodies (Thermo Fisher Scientific, Carlsbad, CA) were also used to evaluate RPE differentiation. F-actin fibers were stained using ActinGreen™ 488 ReadyProbes™ Reagent (Life Technologies, Eugene, OR) and nuclei were stained with NucBlue Live ReadyProbes stain (Life Technologies). After staining, samples were imaged using an LSM 710 Carl Zeiss confocal microscope (Jena, Germany).

### RNA isolation and qRT-PCR

Three and 6 hours after adding mechanical stress, RPE cells from 8 independent cultures (4 unstressed and 4 mechanically stressed) were lysed directly on the membranes and RNA was isolated using an Illustra RNAspin Mini RNA Isolation kit (GE Healthcare, Chicago, IL). A High Capacity RNA-to-cDNA kit (Thermo Fisher Scientific) was used to obtain cDNA from 1 μg of isolated RNA. qRT-PCR was performed using PowerUp™ SYBR Green Master Mix (Thermo Fisher Scientific) in an Eppendorf RealPlex4 real-time Mastercycler (Hamburg, Germany) to measure the expression levels of three *VEGF* isoforms (*VEGF121*, *VEGF165* and *VEGF189*), *HIF-1α*, *ANG2*, *FGF2*, *PEDF*, *IL-6*, *IL-8*, *TNF-α*, *CDH1*, *CDH2*, *VIM*, *RPE65,* and *FN1*. Data were normalized to glyceraldehyde 3-phosphate dehydrogenase (*GAPDH*) values and fold change expression was calculated using the 2^-ΔΔCt^ method.

### Enzyme-linked immunosorbent assay

50 μL samples of spent apical and basal media from control and mechanically stressed RPE cultures were collected after 24 h of applying mechanical stress. The expression of VEGF, ANG2, FGF2, IL-6, IL-8 and TNF-α was tested using a multiplex human enzyme-linked immunosorbent assay (ELISA) kit according to manufacturer’s instructions (Quansys Biosciences, Logan, UT).

### Tube formation assay

Tube formation assays were performed using an in vitro angiogenesis kit (Gibco) according to manufacturer’s instructions. Briefly, the wells of a 48-well plate were coated with 100 μL of reduced growth factor Geltrex matrix (Gibco) and incubated at 37 °C for 30 min. Human umbilical vein endothelial cells (HUVECs) were diluted in spent media from unstressed or mechanically stressed RPE cultures to a concentration of 10^6^ cells/mL. 200 μL of cell suspensions were seeded on Geltrex matrices and incubated for 6 h at 37 °C in a humidified incubator with 5% CO_2_ to induce endothelial tube formation. Next, HUVECs were stained with Calcein AM dye (Thermo Fisher Scientific) and imaged using an Eclipse TS100 fluorescence microscope (Nikon Instrument Inc., Melville, NY). Tube length and node numbers were quantified using the ridge detection plugin for ImageJ software [43, 44].

### Statistical analysis

The data are presented as the mean ± standard deviation (SD) of at least three independent experiments. Comparisons between two groups were analyzed using two-tailed Student’s *t*-test and *p*-values were adjusted using the Benjamini-Hochberg method. *P* < 0.05 was considered statistically significant.

## Data Availability

The datasets generated and analyzed during the current study are available from the corresponding author on reasonable request.
